# Life Habits and Mental Health: Behavioural Addiction, Health Benefits of Daily Habits, and the Reward System

**DOI:** 10.3389/fpsyt.2022.813507

**Published:** 2022-01-27

**Authors:** Hironobu Fujiwara, Kosuke Tsurumi, Mami Shibata, Kei Kobayashi, Takashi Miyagi, Tsukasa Ueno, Naoya Oishi, Toshiya Murai

**Affiliations:** ^1^Department of Neuropsychiatry, Graduate School of Medicine, University of Kyoto, Kyoto, Japan; ^2^Artificial Intelligence Ethics and Society Team, RIKEN Center for Advanced Intelligence Project, Saitama, Japan; ^3^The General Research Division, Osaka University Research Center on Ethical, Legal and Social Issues, Kyoto, Japan; ^4^Integrated Clinical Education Center, Kyoto University Hospital, Kyoto, Japan; ^5^Medical Innovation Center, Kyoto University Graduate School of Medicine, Kyoto, Japan

**Keywords:** habitual behaviours, internet use, media multitasking, resilience, the reward system, motivation, dopamine, exercise

## Abstract

In this review, the underlying mechanisms of health benefits and the risk of habitual behaviours such as internet use and media multitasking were explored, considering their associations with the reward/motivation system. The review highlights that several routines that are beneficial when undertaken normally may evolve into excessive behaviour and have a negative impact, as represented by “the inverted U-curve model”. This is especially critical in the current era, where technology like the internet has become mainstream despite the enormous addictive risk. The understanding of underlying mechanisms of behavioural addiction and optimal level of habitual behaviours for mental health benefits are deepened by shedding light on some findings of neuroimaging studies to have hints to facilitate better management and prevention strategies of addictive problems. With the evolution of the world, and the inevitable use of some technologies that carry the risk of addiction, more effective strategies for preventing and managing addiction are in more demand than before, and the insights of this study are also valuable foundations for future research.

## Introduction

Dopamine (DA) is a critical neurotransmitter for maintaining and promoting favourable human mentality, including healthy mood and motivation, through its function within the reward/motivation system. On the other hand, the relationship between addictions, such as cocaine addiction, food addiction, and the problematic use of the Internet (PUI), one of the recent types of behavioural addiction, and the deficits of the reward system have been widely demonstrated by neuropsychological studies, including neuroimaging studies (1–3).

Excessive behaviours, which characterise behavioural addiction, as well as substance addictions, are thought to be underpinned by a dysregulation of the DA reward/motivation system, which is typically based on the reward deficiency syndrome (RDS) hypothesis (4, 5). The RDS hypothesis supports the innate susceptibility to addictive problems; for example, genetic polymorphisms of DA-related receptor genes are associated with various behavioural addictions, including hypersexuality, gambling disorders, and Internet gaming. In addition, impulsive behaviour-related disorders, including autism and attention-deficit hyperactivity, are involved in the concept of RDS (5).

It needs to be ascertained if environmental factors influence the development of the deficiency in the neurotransmission of the reward-related circuitry. Several positron emission tomography (PET) studies have shown that postsynaptic DA receptor change/downregulation occurs as a consequence of high-level DA release induced by conditions such as sleep deprivation, nicotine abstinence, and PUI (6–8). It also needs to be determined whether the activation of the reward/motivation system, namely the DA system, is associated with health benefits or issues when the level is low to intermediate.

In this review article, we shed light on the health benefits/negative aspects of daily behaviours, including up-to-date life habits, Internet use (IU) and media multitasking, physical exercises, gambling, and related psychological resilience, and their potential neural correlates in the reward/motivation system.

## Neural Correlates of Dopamine Neurotransmission, the Reward System, and Their Association With Motivation and Resilience

**Figure 1:**
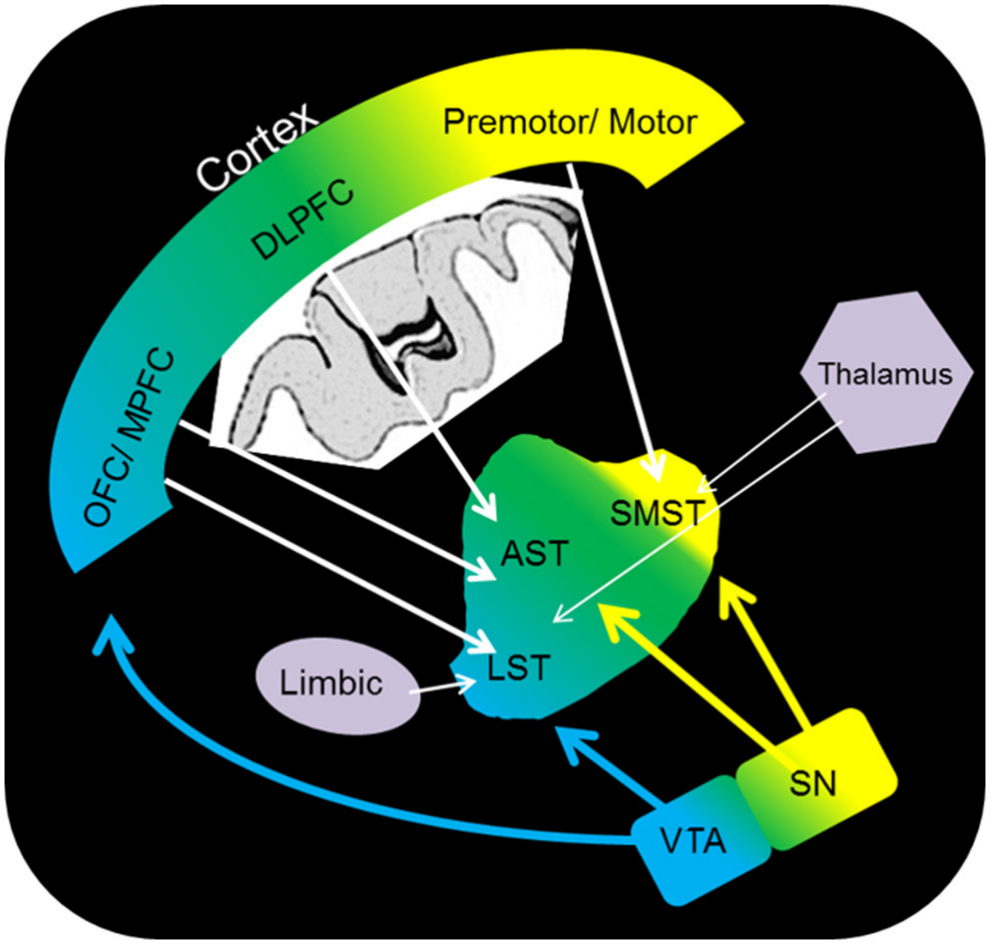
Schematic representation of the dopamine pathway. OFC, orbitofrontal cortex; DLPFC, dorsolateral prefrontal cortex; premotor/motor, premotor/motor cortex; SMST, sensorimotor striatum; AST, associative striatum; LST, limbic striatum; VTA, ventral tegmental area; SN, substantia nigra.

The reward system is a well-characterised network of regions across the limbic, prefrontal, striatal (with functional subdivisional separation: associative, sensorimotor, and limbic striatum), and midbrain regions (Figure 1). Neuroimaging studies suggest that the key structures in this network are the anterior cingulate cortex, the orbital prefrontal cortex, the dorsal prefrontal cortex, the limbic striatum, the ventral tegmental area (VTA), the thalamus, and the midbrain DA neurons. DA is a well-established reward system neurotransmitter; DA neurons are present throughout regions within the midbrain, including the VTA and limbic striatum, and reward-related stimuli induce DA release (9). The relationship between the reward system and cognitive/affective/psychological terms closely overlaps with “motivation”, and key regions of the system consist of “the motivation network” (10), which involves the regions activated by a reward-related task (11). Several reports have suggested a relationship between the reward system and “resilience”. In animal studies, DA antagonist administration has been shown to increase physiological stress responses (12), and DA injection to the central nucleus of the amygdala induces attenuation of stress-related ulcer formation (13), which suggests the role of DA in terms of stress regulation. In this context, one possible interpretation is that DA is considered to be associated with “motivation” and “resilience”, which is critical as a background trait for establishing stress coping strategies of individuals (14).

## The Association of the Motivation and Reward Network With Internet Use

IU is a widespread and unique feature of modern human society. Several aspects of daily life have been facilitated dramatically by IUs. In recent years, evidence has suggested that the PUI influences mental status (15). PUI is now regarded as a behavioural addiction, and one of the predictive frameworks for PUI addiction pathology, as well as other addictions, is the RDS hypothesis (4, 5), which proposes a reduction in the DA neurotransmission/attenuated reward system based on the results of PET studies (reduced DA D_2_ receptor availability) (8, 16) and functional magnetic resonance imaging (fMRI) studies (lower brain activity of the mesolimbic pathway) (17). Amongst the various forms of IU, problematic Internet gaming, or Internet gaming disorder (IGD), has been included within the framework of the standard diagnostic criteria of the International Statistical Classification of Diseases and Related Health Problems-11 (ICD-11). The purposes of IU are diverse and may constitute the actual problem rather than IU itself. Recent structural neuroimaging studies have demonstrated changes in the cortical regions that are involved in the reward system in IGD (18–20), as well as the changes in white matter fibre tracts (21). These MRI findings are also consistent with positron emission tomography (PET) observations of decreased DA receptor availability in the striatum in IGD (16). FMRI studies (both during resting state and reward-related paradigms) have also demonstrated the involvement of reward-system deficiency in individuals who frequently play online games (17, 22, 23). Taken together, these findings from structural and functional imaging studies indicate that reward system function is attenuated in IGD.

Although pathological deficits of the reward system are being elucidated for their association with behavioural addiction, including PUI, it still cannot be determined whether IU always harms mental health/cognitive function, even for cases of low to intermediate use. We can no longer avoid the online environment in daily life activities, including learning and work. This feature distinguishes PUI from other specific addictive behaviours, such as gambling and alcohol drinking. Therefore, it is unique and worthwhile to assess the optimal daily IU based on its health effects. For example, a structural MRI (voxel-based morphometry) study revealed that online social networking service size, typically represented by the number of online friends, was positively associated with regional brain volumes in healthy individuals (24), indicating that IU for social interaction may affect the brain positively. Clinically, treating various kinds of comorbid psychiatric disorders, such as attention-deficit hyperactivity disorder, depression and anxiety, obsessive-compulsive disorder, and autism spectrum disorder (ASD), is critical for PUI therapy, in addition to the direct intervention of PUI *per se* (25–28). Therefore, it is important to consider the effects of comorbid features of PUI in investigating its benefits.

An fMRI study by Fujiwara et al. (29) was conducted to investigate the neural correlates of clinically insignificant (low to intermediate) IU based on the reward/ motivation network function of 121 healthy volunteers, focusing on ASD tendency as a comorbid feature of IU since IU with ASD tends to lead to the “Hikikomori” situation, particularly in young people (29). The extent of IU was evaluated using the Generalised Problematic Internet Use Scale 2 (30), which consists of 15 questions (five subscales: “Preference for Online Social Interaction,” “Mood Regulation,” “Compulsive Use,” “Cognitive Preoccupation,” and “Negative Outcomes”). The Deficient Self-Regulation subscale scores correspond to the summation of the compulsive use and cognitive preoccupation scores.

For the MRI dataset analysis, a region of interest (ROI)-to-ROI FC analysis was applied to the resting-state fMRI scans using the CONN-fMRI Functional Connectivity toolbox (ver.17e) with the statistical parametric mapping software package SPM12 (Wellcome Trust Centre for Neuroimaging, http://www.fil.ion.ucl.ac.uk/spm). Twenty-two spherical clusters (10-mm diameters) were specified and peak coordinates based on a previous motivation-related fMRI study (10). Briefly, the ROIs of the motivation network were located in the medial prefrontal cortex, supplementary motor area, intraparietal sulcus, frontal eye field, inferior parietal lobule, middle frontal gyrus, midbrain, caudate, putamen, nucleus accumbens, rostral anterior cingulate cortex, anterior insula, and precentral gyrus. The associations between the GPIUS2 scores and the association between the functional connectivity (FC) values of the two ROIs in the motivation network were explored using CONN for FC analysis (age and gender as covariates, *p* < 0.05). Mediation analysis was performed to investigate whether autistic traits (indexed by the autism spectrum quotient (AQ) mediated the association between the degree of IU and FC values of the network. A nonparametric bootstrap method (2000 bootstrap samples) was used to test the mediation path (indirect effect of GPIUS2 scores on FCs through a mediator, i.e., AQ scores).

**Figure 2:**
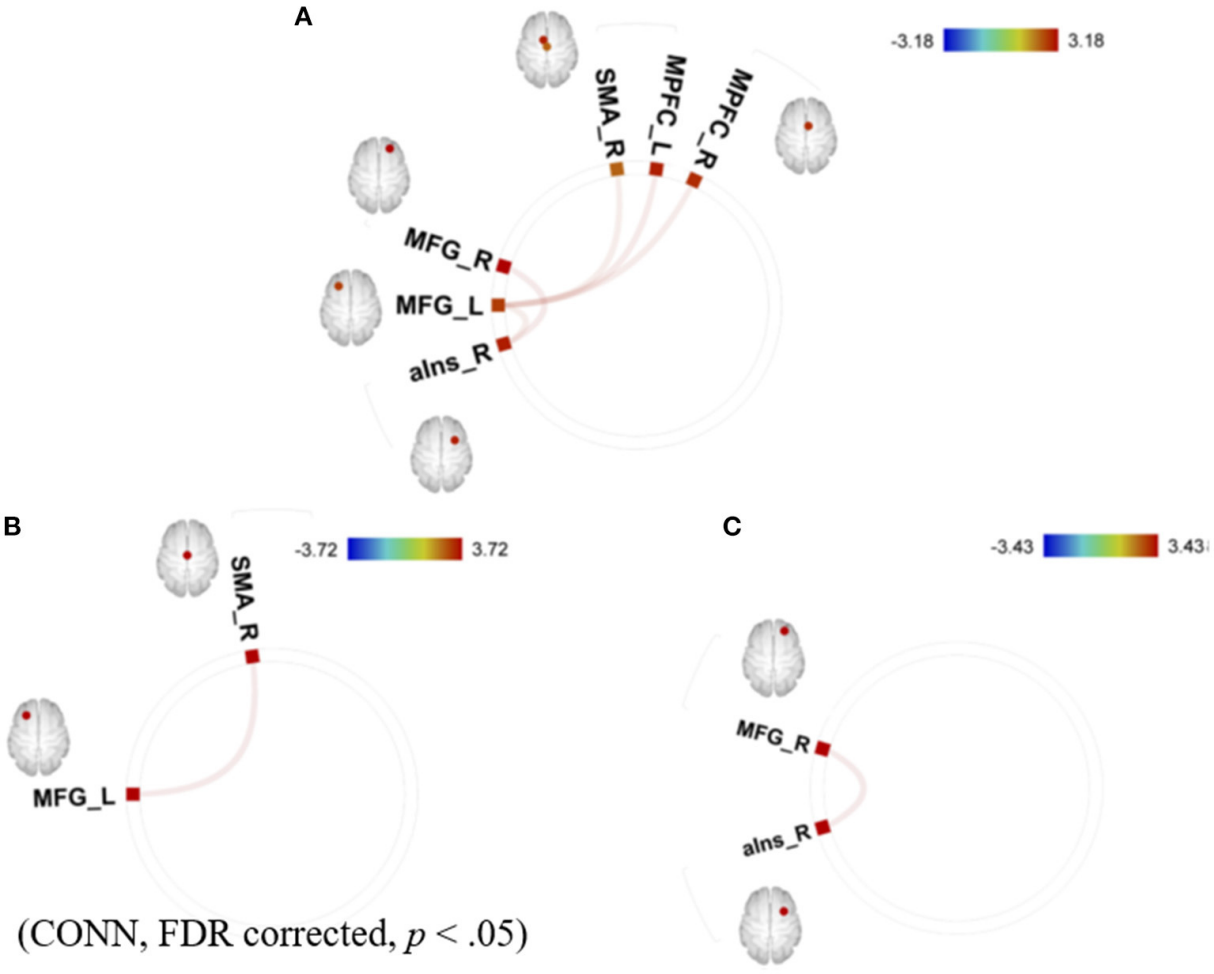
Correlations between **(A)** total GPIUS2 scores, **(B)** the scores of mood regulation, and **(C)** the scores of deficient self-regulation and functional connectivity of the motivation network. MPFC, medial prefrontal cortex; SMA, supplementary motor area; MFG, middle frontal gyrus; aIns, anterior insula; L, left; R, right. The colour bar indicates the positive (red) and negative (blue) *T*-values for the correlations [Revised from Fujiwara et al. (29)].

**Figure 3:**
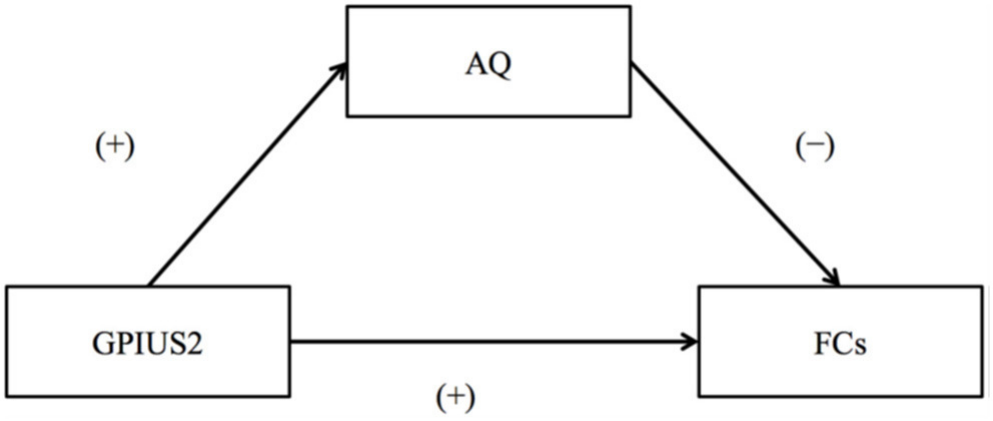
The relationships among Internet Use, Autistic Traits, and Functional Connectivity of the motivation network. (+) and (–) indicate the direction of correlation coefficients for GPIUS2 and FC, GPIUS2 and AQ, and AQ and FC, respectively. AQ, autism spectrum quotient; FC, functional connectivity correlated with GPIUS2 scores; GPIUS2 scores; GPIUS2, generalised problematic internet use scale 2 [Revised from Fujiwara et al. (29)].

The summary of the results is as follows: the mean GPIUS2 scores in the study were similar to those in a previous study of healthy young individuals (23), suggesting subclinical level (36.2 [15.7]). The AQ scores were also within a subclinical level. Correlations between the GPIUS2 total scores and those of the “mood regulation” subscale scores and the FCs within the motivation network are shown in Figure 2. Briefly, the GPIUS2 was positively associated with the FCs of the motivation network. A representation of the association among GPIUS2, AQ, and FC is shown in Figure 3. The AQ scores were negatively correlated with the FC, whereas total GPIUS2 scores and those of subscales were positively correlated with both FC and AQ scores. For the mediation analyses, the bootstrap method revealed a significant mediation effect of autistic traits, indicating a negative impact on the relationship between GPIUS2 and FC.

The results revealed a positive association between the IU and FC of the reward/motivation network. The findings of the past PUI studies were generally consistent with the attenuated reward-system functions, such as the decreased D_2_ receptor availability (8, 16) and the weakened brain activation during anticipation of monetary rewards in the limbic striatum (17) and reduced resting-state FC of mesolimbic reward pathway (3). The positive association between the IU and FC in low-to-moderate users indicates that subclinical IU may play a role in maintaining the reward/motivation-network integrity. The GPIUS2 scores were positively correlated with the AQ scores and negatively correlated with FC (Figure 3). Mediation effects of autistic traits in mediation analysis indicated that even subclinical level autistic traits may negatively impact the effects of IU on motivation. Individuals with autism have difficulty carrying on with their daily lives, including communicating with others without IU. Ueno et al. (31) suggested the possible benefits of mental health support and interventions as strategies for young medical residents, considering the risk of PUI and the potential prevalence of subclinical autistic traits (31). IU (as far as it is at an adequate level) may help autistic individuals, including those with the Hikikomori situation, by offering a safe place in which they can communicate with others (32). Therefore, approaches to encourage safe and adequate IU should be considered (instead of seeking a way to prohibit it completely), for example, in the context of school education.

## Reward and Media Multitasking

In recent years, various types of media have been used, particularly by the young generation. The young generation simultaneously uses several media gadgets, which is termed media multitasking (33).

The potential risks of media multitasking are thought to be present because of its association with other habitual behaviours that may lead to addictive problems. For example, PUI (mentioned above) may be implicated in media multitasking because it may include several forms of IoT usage, such as different kinds of social media, online gaming, shopping, and pornography at the same time. Another unique example of a comorbid problem of multitasking is obesity (34, 35); it is one of the major public health issues and is often referred to as “food addiction” (2), which is explained by its association with lower DA functioning. Lopez et al. (34) presented a unique hypothesis that heavy level media multitasking is associated with greater reward sensitivity, that is, sensitivity to external food cues. In their study, excessive multitasking was associated with a higher risk of obesity. Furthermore, functional imaging during exposure to appetitive food cues showed changes in regional activity to food cues, specifically with an imbalance favouring reward-related recruitments in the limbic striatum and the orbital prefrontal cortex, which are involved in the mesolimbic pathway of the reward system (34). Taken together, findings from the study suggest excessive media multitasking as a behavioural addiction underlaid by the alteration of reward-related DA neurotransmission, which is associated with the potential risk of food over-intake.

Concerns have been raised about the impact of media multitasking on attention and two opposing hypotheses have been provided: the scattered attention hypothesis and the trained attention hypothesis (36). On the one hand, the scattered attention hypothesis means that continuous media multitasking gives negative impacts on attention control and maintains the focus on target issues. On the other hand, the trained attention hypothesis corresponds to the positive effects of multitasking on cognitive control during attention processing.

Several studies have suggested that a higher cognitive load for switching between tasks is needed for heavy multitaskers (37, 38), which supports the scattered attention hypothesis. On the other hand, Alzahabi et al. (36) reported that media multitasking contributed to better task-switching performance (36), which supports the trained attention hypothesis. Thus, these findings are inconsistent in the direction of their influences on attention processing.

In an fMRI study by Kobayashi et al. (39), the association between focused attention and low-to-intermediate multitasking lifestyles was explored in 103 participants (66 men, 29 ± 11.6 years) using the Media Multitasking Index (MMI) as an index of multitasking (39). This study focused on participants presumed to engage in low-to-intermediate multitasking. Regarding the quantification of multitasking. The network of interest in the study was the dorsal attention network (DAN), which involves focused attention and its goal-directed top-down regulation. The number of FCs with strengths surpassing a threshold was assessed as an intra-network connectivity marker (Degree Centrality. DC). The DCs of the DAN for the resting state and the auditory oddball paradigm that required focused attention processing were compared. The relationships between MMI and DCs were explored at each resting and task phase.

**Figure 4:**
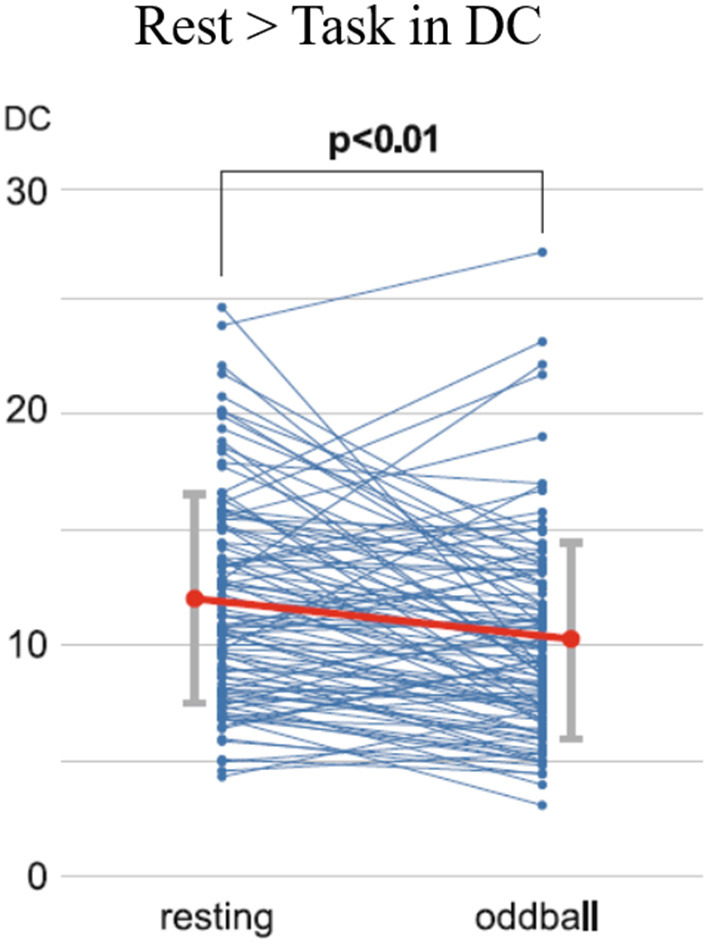
Time-course changes of the DCs for the resting state and oddball task phase. Each dot indicates the DC values for each participant. A significant decrease was found in the DCs from the resting state to the oddball task phase, as calculated using paired *t*-tests (*t* = 4.56, *p* < 0.01). DC, degree centrality [Revised from Kobayashi et al. (39)].

**Figure 5:**
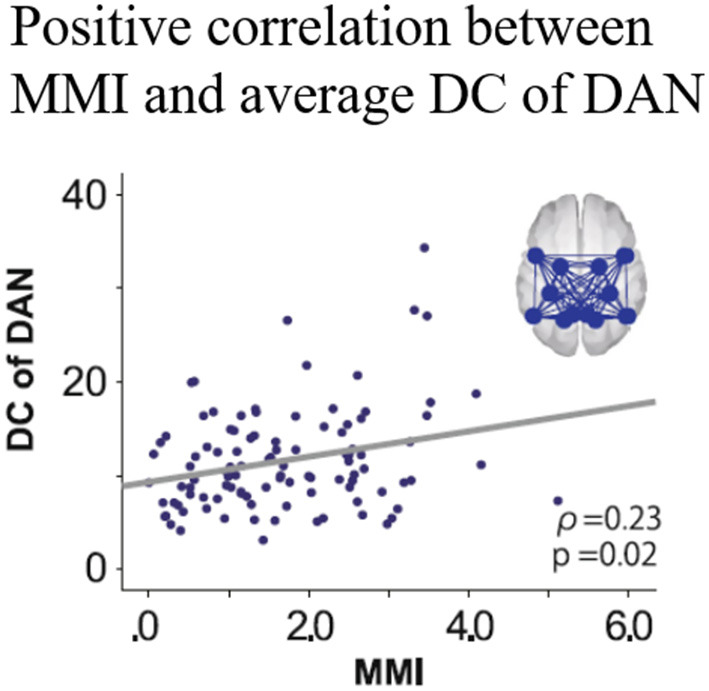
Correlation between MMI and DCs of the DAN during the oddball task. MMI, media multitasking index scores; DC, degree centrality; DAN, dorsal attention network [Revised from Kobayashi et al. (39)].

The mean MMI (1.68) was lower than the scores reported by previous studies, indicating that the extent of multitasking was low to intermediate but pathological among the participants of the study [cf. Ophir et al. (37): 4.38; Alzahabi and Becker. (36): 4.07; Cardoso-Leite et al. (40): 3.98]. The DCs within the DAN for the task phase were lower than those during the resting state (Figure 4). Correlation analyses showed no significant correlation between the MMI and DCs during the resting state, while the MMI-DC association was present during the task (Figure 5).

Considering the results of the comparison of the DCs between the rest and the task phase and the positive correlation between MMI and the DCs during the task, DC reduction from the resting state to the oddball task was interpreted to be attenuated in higher media multitaskers than in those with lower levels. An fMRI study suggested that higher performance during a cognitive task is associated with smaller changes in FCs between the resting state and task conditions (41). In this context, smaller DC changes in greater multitaskers would be interpreted to be more suitable for the attentional demands, indicating “trained attention processing” during past multitasking of the higher multitaskers. Interestingly, one study reported that intermediate level media multitaskers performed better on attentional tasks compared to both light and heavy multitaskers (41), suggesting a possible inverted U-shaped relationship between media multitasking and focused attention. If this tentative model was accepted, the participants in the study are located near the centre of the inverted U curve, resulting in better attention performance in intermediate level multitaskers. This interpretation supports the trained attention hypothesis, but it is restricted to the cases of light to intermediate multitaskers. Further studies would be needed to clarify the validity of the “inverted U shape” model, comparing the relationship between multitasking tendency and attention function among various multitaskers including heavy multitaskers.

## BUDO and the Motivation/ Reward

Budo is a term in Japanese martial arts such as Judo, Karate, Kendo, and Aikido. Budo is often regarded as a sport; one of its unique characteristics is the emphasis on both the mind and body because its tradition has been partially adapted from concepts of Zen Buddhism, like the recent mindfulness meditation (42). Budo emphasises the importance of a calm, unmoving, and undisturbed mind; these aspects are described as Fudoshin (unmoving mind) or Mu (empty mind; Oosterling) (43). In contrast with the contemplative meditation of the sitting Zen, Budo is regarded as “Zen in action” (43), and physical training is an essential component.

The benefits of physical exercise can be experienced empirically in our daily lives, typically by walking. Several reports have suggested that engagement in sports positively influences mental health. Habitual exercise (at least a few times a week) has been found to alleviate depression and anxiety, foster the Sense of Coherence (which is closely associated with stress coping), and improve self-esteem and cognitive functions (44). The benefits of sports on cognitive function have been reported in meta-analyses (45, 46); for example, an intermediate level of aero bike pedalling contributes to the improvement of memory function (47). Kida et al. (48) found that professional baseball players had shorter reaction times (RTs) to target stimuli during an attentional task. Furthermore, in the same study, 2-year longitudinal follow-up showed further shortening of RTs, indicating positive influences of the training on performance (48).

**Figure 6:**
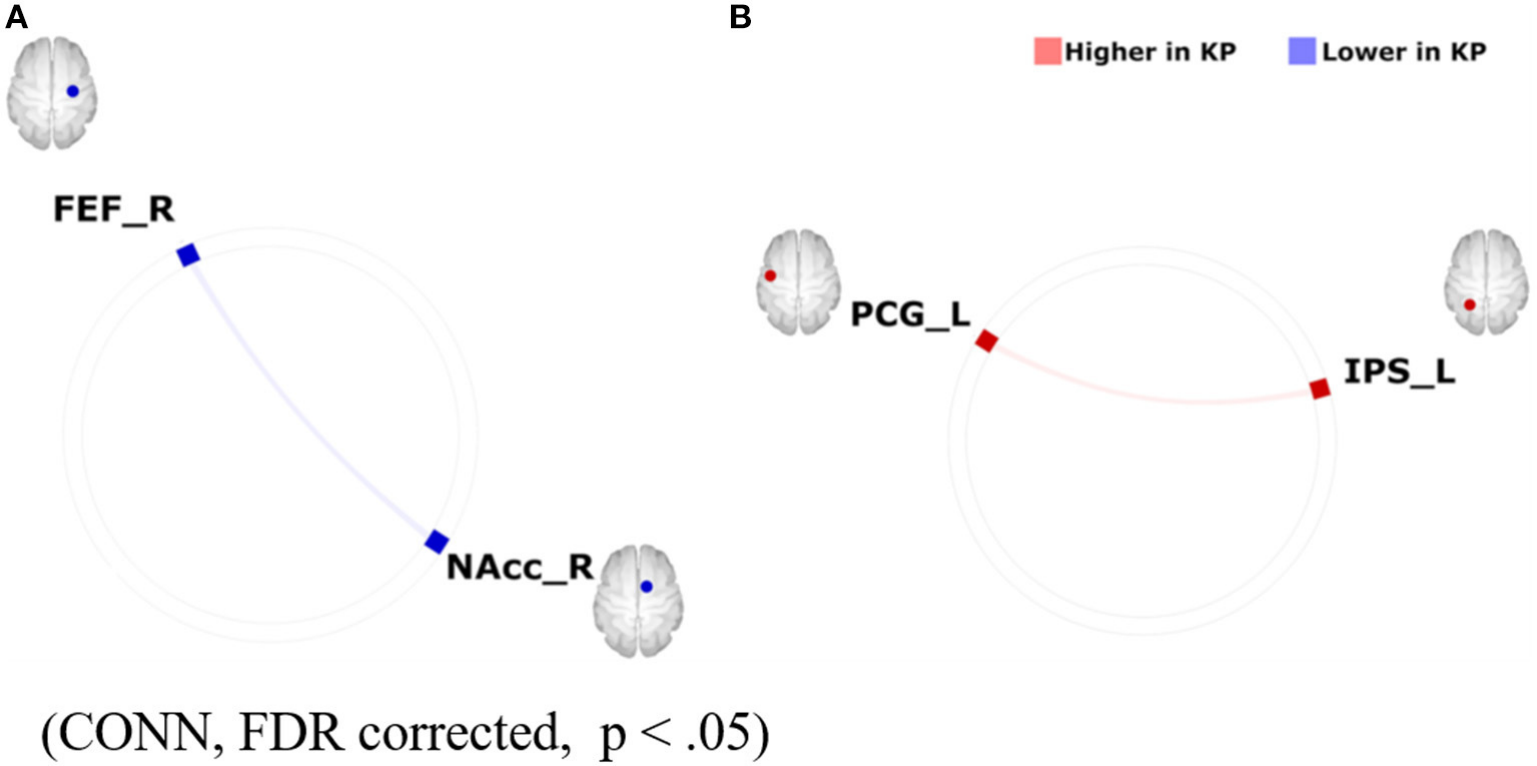
Group differences in FC within the motivation network on **(A)** resting-state-functional magnetic resonance imaging (rs-fMRI) and **(B)** task-based fMRI during an auditory oddball paradigm (KPs < NKPs/ KPs > NKPs). FEF, frontal eye field; FC, functional connectivity; NAcc, nucleus accumbens; PCG, precentral gyrus; IPS, intraparietal sulcus; L, left; R, right; KP, Kendo players; NKP, Non-kendo players [Revised from Fujiwara et al. (54)].

Despite evidence of the positive impact of the mind-body integrated training on cognitive functions such as attention (49–51), the underlying neural mechanism is not yet well-known. Here, one possible perspective is that investigating the impact of Budo on reward/motivation, which is closely associated with mental illnesses typically depression, can help to address this issue since cognitions are thought to be influenced by motivation (52). An example that suggests the emphasis of motivation in Budo is found in “The purpose of practising kendo” (https://www.kendo.or.jp/knowledge/kendo-concept/: All Japan Kendo Federation, 1975), which includes the one “*to cultivate a vigorous spirit* through correct and rigid training”. A calmness in mind, as well as instantaneous concentration, are essentially needed in establishing higher performance in Budo. According to the Drive Theory (53), motivation is needed to provide the energy to trigger, maintain, and direct goal-related behaviours, influencing our daily behaviours. Regarding the neural correlates of motivation, the motivation network (10), (as introduced in the section “The association of the motivation and reward network with Internet Use” in the current review) has ROIs consisting of the brain regions that are involved in the major part of the reward system. One possible hypothesis is that the ability of “switching” between the resting and attentionally-driven states of motivation is fostered and the ability gets more effective through the mind-body training of Budo. To address this, Fujiwara et al. (54) focused on one of the Budos Kendo players with Dan-grade (that is, a highly-skilled and long-term habitual player), considering the characteristic of extremely fast movement during competition with opponents (54). An fMRI study on the motivation network was conducted in 14 Kendo players (KPs) and 11 non-KPs (NKPs) during the rest and the auditory oddball task. FCs were calculated using CONN-software within the motivation network. The authors also assessed several major confounding factors such as body mass index, habits of alcohol drinking and smoking, an index of general physical activity, International Physical Activity Questionnaire (IPAQ) scores, and reaction time during the oddball task. As result, KPs were lower than NKPs in FC between the right frontal eye field and the right nucleus accumbens within the motivation network during resting state. In contrast, KPs were higher than NKPs in FC between the left precentral gyrus and the left intraparietal sulcus during the auditory oddball task (Figure 6). These differences in FC remained significant after controlling for major confounding variables. The results suggest that the contrast between the enhanced motivation network integrity under attentional demands and those attenuated during rest may underlie the abilities of instantaneous concentration in KPs, which are presumably cultivated by the continuous habitual training of Kendo. Finally, although the result of the study by Fujiwara et al. (54) supports the favourable effects of Kendo on motivation, excessive sports training, including Kendo, may lead to negative mental health consequences, typically as represented by “overtraining syndrome” (55) or sports addiction (56). Considering the potential negative and positive effects of exercise on mental health, further studies should be conducted to determine the appropriate quantity, quality, frequency, and intensity of training. Kendo is practised by over 2.5 million people worldwide, a number that is suitably large to allow us to generalise the importance of the mindset of Budo. Furthermore, practitioners of Judo, another major Budo, can be found in large numbers across the world (including 16,000 in Japan and several million worldwide) (International Kendo Federation, 2014: The Nikkei online (2018). Available online at: https://www.nikkei.com/article/DGXMZO87539520R00C15A6000000/). Thus, if we recognise the commonality of the concepts adopted by its different forms, we would be able to generalise the mental health benefits of Budo. However, the outcomes reported by Fujiwara et al. (54) cannot explain the Kendo-specific positive effects on motivation. Future studies would be needed to determine the presence of Budo-specific mental health benefits in relation to various cognitive functions, including motivation, with direct comparisons among different disciplines.

## Resilience, Attention, and the Reward System

Resilience refers to the capacity to adapt to acute stress, chronic adversity, or trauma (57). It can be thought of as “bouncing back” from a difficult experience. Human responses to stress vary widely in severity and manner. Some people develop stress-related disorders, such as posttraumatic stress disorder and depression, whereas others demonstrate no symptoms due to stress. Therefore, clarifying the neural mechanisms of resilience can potentially lead to finding a biomarker for the risk of the occurrence of or transition to stress-related psychiatric disorders. Evidence suggests a critical role of the reward system in modulating the fight-or-flight stress response in ways that confer stress resilience. For example, pharmacological studies have highlighted that reward system neurotransmitters, including DA, play a critical role in stress resilience. DA is known to foster stress resistance by preventing exaggerated behavioural and physiological stress reactivity and is associated with active coping (58). Notably, in an animal study, DA administered to the central nucleus of the amygdala prevented stress-induced ulcer formation (13), suggesting a stress regulation by DA [reviewed in Dutcher et al. (14)].

Regarding the neural correlates of reward and resilience (stress responses), both are mutually suggested to overlap (59). As the medial prefrontal cortex and basolateral amygdala, both of which are involved in the reward circuitry, regulate stress responses, they are likely to regulate the hypothalamic-pituitary-adrenocortical axis and autonomic nervous system responses to both reward stimuli.

Miyagi et al. (60) attempted to investigate attention demands such as “stress” and examine the relationship between attentional loads and resilience, focusing on functional connectivity of the default mode network (DMN) (60). The DMN is considered to maintain basic cognitive processes such as those associated with anticipation processes to plan for the future, self-awareness, and consciousness (61). One study suggested that resilience is associated with the ability of attentional control in healthy individuals (62). FCs of the DMN have been suggested to associate with resilience negatively in healthy individuals during the resting state (63). A questionnaire, the Connor–Davidson Resilience Scale (CD-RISC), was used to assess the level of resilience (the higher the scores, the higher the resilience). fMRI scans were used during the resting state and the oddball task to investigate the relationship between resilience in healthy participants (*N* = 89, age = 32.1 years, male/ female = 59/30) and changes in FC of DMN during “switching” (from “rest” to “task” phase) and “sustaining” (task phase in which cognitive efforts are demanded continuously). The reaction time was used as an index of the performance of the paradigm. The parameters were assessed for the two-time windows separately: (1) 180 seconds at the former half of the task [“Odd 1”] and (2) the latter 180 s of the task [“Odd 2”]. As for the relationship between task performance and resilience, no correlation was found between the reaction time and the CD-RISC scores. In addition, task performance did not differ between the low (CD-RISC score ≤ 58) and high (CD-RISC score ≥ 59) resilience groups.

**Figure 7:**
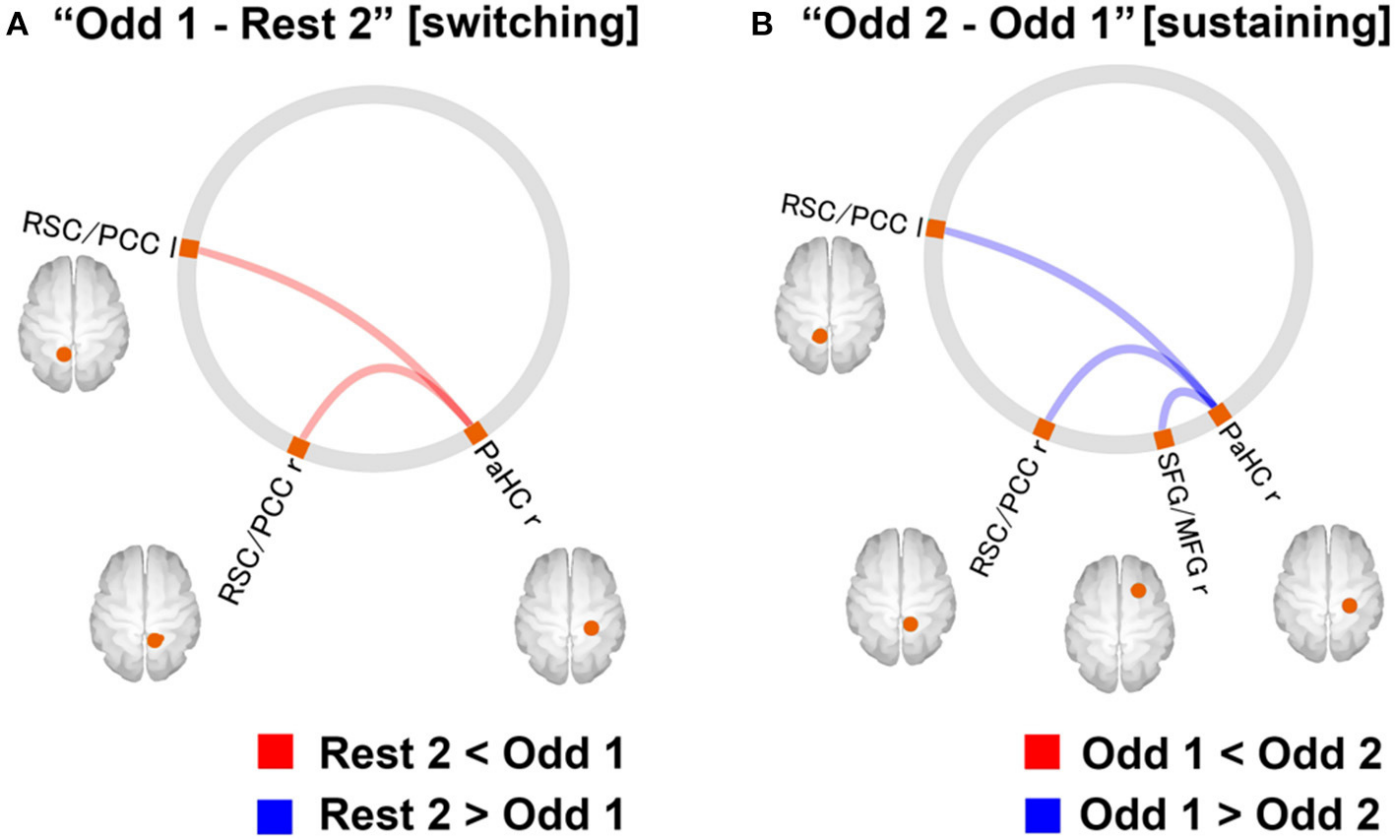
**(A)** Changes in DMN-FC at “switching” phase. The red line indicates that the FC values for the “Odd 1” period were significantly higher than those for the “Rest 2” period. **(B)** FCs with a significant difference at “sustaining” phase. The blue line indicates that the FC values for the “Odd 2” period were significantly lower than those for the “Odd 1” period (statistical threshold, FDR corrected, *p* < 0.05). FC, functional connectivity; FDR, false discovery rate; DMN, default mode network; r, right; l, left; PCC, posterior cingulate cortex; RSC, retrosplenial cortex; SFG, superior frontal gyrus; MFG, middle frontal gyrus; PaHC, parahippocampal cortex [Revised from Miyagi et al. (60)].

**Figure 8:**
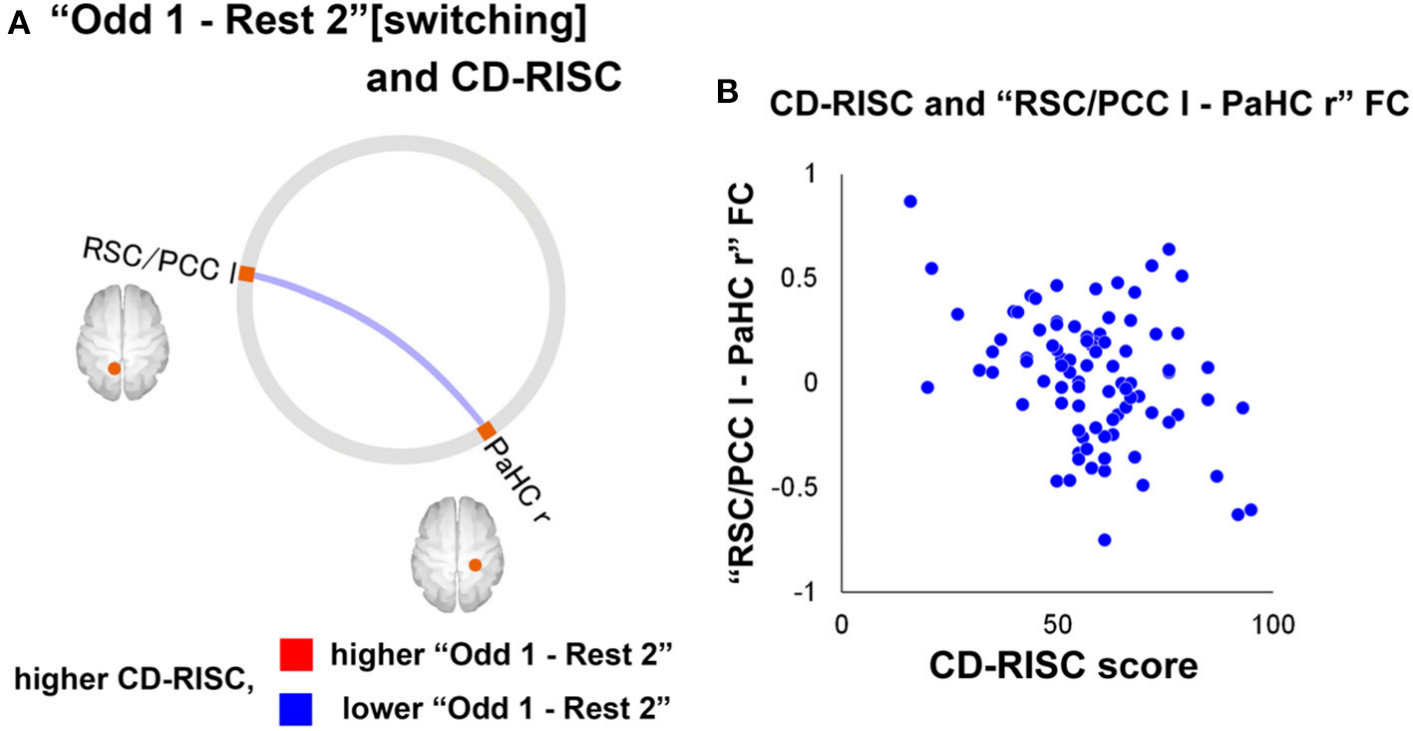
**(A)** A significant correlation between CD-RISC scores and the FC changes at “switching” phase. The blue line indicates that there was a negative correlation between the value for the “Odd 1–Rest 2” period and CD-RISC scores. **(B)** Scatterplot of the relationship between CD-RISC scores and the “RSC/PCC l-PaHC r” FC for “Odd 1–Rest 2”. CD-RISC, connor–davidson resilience scale; DMN, default mode network; ROI, region of interest; r, right; l, left; lr, left and right; PCC, posterior cingulate cortex; PaHC, parahippocampal cortex; RSC, retrosplenial cortex [Revised from Miyagi et al. (60)].

Neuroimaging data were analysed using the CONN software. The preprocessed fMRI time series in the ROIs of DMN were extracted for four-time windows (180 s each): (1) the former half of the resting state [“Rest1”], (2) the latter half of the resting state [“Rest 2”], (3) [“Odd 1”], and (4) [“Odd 2”]. Significant differences in FCs between [switching] “Rest 2” and “Odd 1” and “Odd 1” and “Odd 2” [sustaining] are shown in Figure 7. Regarding the FC difference at the “switching” phase, “Odd 1” was significantly higher than “Rest 2” in FC values between the right parahippocampal cortex (PaHC) and the posterior cingulate and bilateral retrosplenial cortices (Figure 7). As for the difference at the “sustaining” phase, “Odd 2” was lower than “Odd 1” in FC values between the right PaHC and the bilateral retrosplenial and posterior cingulate cortices, as well as those of the FCs between the PaHC and the superior and middle frontal gyrus (Figure 8). In correlational analysis, the CD-RISC was negatively correlated with the changes in FCs between bilateral retrosplenial and posterior cingulate cortices during “switching” (Figure 8).

The increased DMN FCs in the study may be explained as follows: (1) In general, the regions consisting of the DMN are deactivated by performing any cognitive tasks (64, 65). However, the activity may increase during tasks if they are relatively easy and needs a less cognitive load to perform. (2) The regions of DMN, such as the cingulate and frontal gyrus, play roles during the auditory oddball task (66). A decreased FC at the “sustaining” phase corresponds to mental fatigue (67). A negative correlation between the increase in FC during “switching” and CD-RISC may be interpreted as follows: The increase in FCs during the switching phase was attenuated in high resilience individuals, whereas heightened in those with low resilience, indicating homeostatic DMN-FC in high-resilience individuals. In this context, DA may be a critical neurotransmitter in maintaining homeostatic DMN FC from the perspective of its potential impact on fostering resilience.

## Dopamine and Gambling Disorder

In addition to clinical similarities with substance use disorders (SUD), accumulating evidence of biological similarities has led to the classification of gambling disorder (GD) in the same category as substance use disorders in the DSM-5 (68). However, regarding DA, the similarities are not straightforward. Parkinson's disease patients with DA agonist treatment sometimes show increased reward-seeking behaviours, such as gambling disorder (69), and it seems reasonable that DA can influence the development of gambling disorder, but the results of the PET study challenge this inference. PET studies of SUD (70) and other behavioural addictions such as food addictions (6) have generally found reduced D_2_ receptor availability in the striatum, which supports the RDS hypothesis. However, in PET studies of GD patients, D_2_ receptor availability in the striatum did not differ from that in the healthy group (71). Taken together, the RDS hypothesis can explain the behaviour of SUD patients, but it cannot always explain the behaviour of patients with GD.

MRI studies can provide explanations for this problem. There is a widely used task called the monetary incentive delay task (MIDT) (72). MIDT is inspired by the epoch-making primate study that showed a shift in dopaminergic activity in the VTA from the time of reward delivery to the time of cue presentation, which predicts reward according to the acquisition of Pavlovian conditioning (73). Addiction patients, including GD patients, are repeatedly exposed to rewards, and the measurement of brain activity is necessary during reward prediction. Thus, MIDT and its variants are applied to the addiction population to measure activity in the striatum, which is the target region of VTA dopaminergic neurons, during reward anticipation. In MIDT, a cue to anticipate reward is presented. Next, some actions (e.g., button press in a limited time) are needed for participants, and a specific reward is presented afterward. Brain activation during the period from cue presentation to reward delivery was analysed as activation during reward anticipation.

In a meta-analysis of MIDT, both GD patients and SUD patients showed reduced striatal activation during reward anticipation (74). This result may complement the RDS hypothesis in patients with GD. Additionally, the effect of cues and rewards presented in the MIDT may confer another complementation. Two studies utilised the monetary cues depicted in simple words. GD patients in these studies showed lower striatal activation than healthy controls (HCs) during reward anticipation (75, 76). Tsurumi et al. (77) performed an fMRI study adopting a paradigm using symbol cues that correspond to a specific number of points to be earned if participants could respond in time (77). GD patients (Japanese slot machine gamblers) in this study and the HCs showed comparable striatal activation. One study used “$” symbols and erotic silhouette symbols as cues that precede monetary and erotic rewards, respectively. Relative to the HCs, the GD patients in this study showed comparable striatal activation during monetary reward anticipation and decreased striatal activation during erotic reward anticipation (78). One study used playing card stimuli as cues, and the GD patients showed increased striatal activation during reward anticipation (79). Summarising the above, GD patients showed decreased striatal activation following simple word monetary cues and erotic silhouette symbol cues, comparable striatal activation following “$” and point indicating symbol cues, and increased activation following addiction-specific cues. In other words, striatal activation of GD patients during reward anticipation was modulated by cue presentation, such that the similarity of cues for an addictive object increases activity. Moreover, this phenomenon may account for the vicious cycle of GD patients who have tremendous interest in gambling-related activity and cannot take pleasure from other activities.

**Figure 9:**
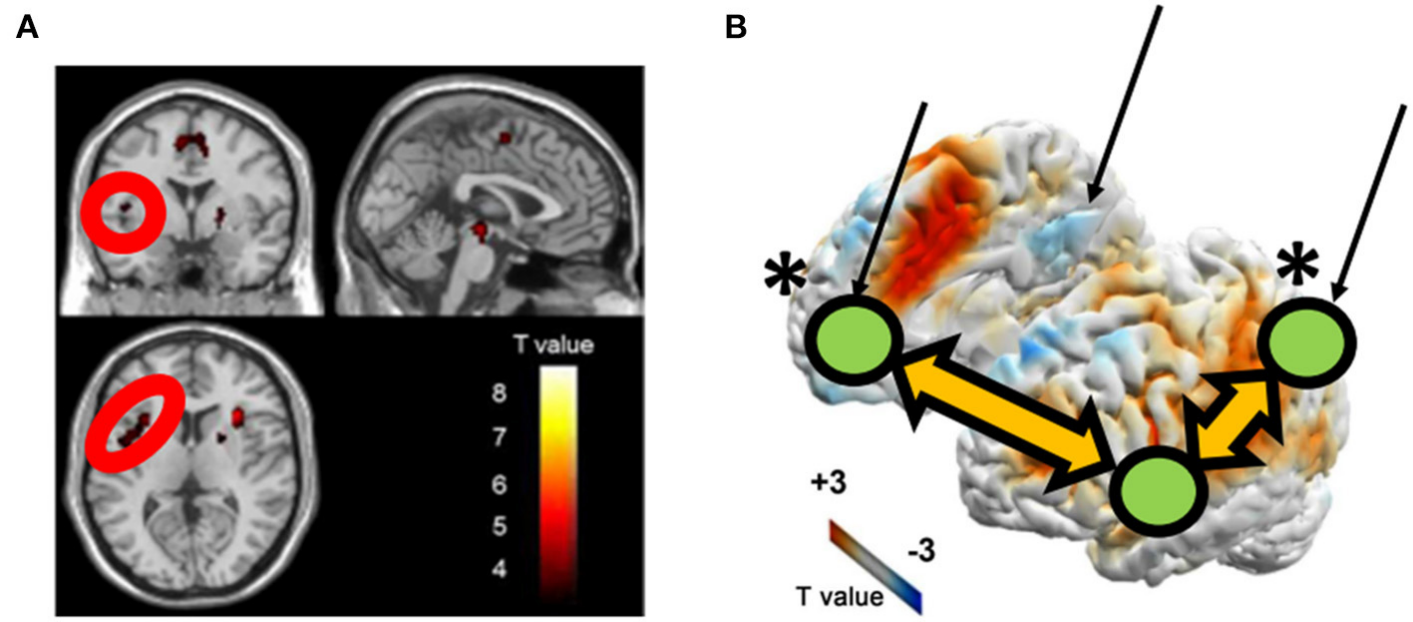
Brain activity **(A)** and functional connectivity **(B)** are associated with the duration of illness in patients with gambling disorder. **(A,B)** is revised from Tsurumi et al. (77) and Tsurumi et al. (83), respectively. The arrows indicate the locations of DMN regions. The *indicates a between-group difference regarding the insular seed and marked DMN nodes.

In addiction literature, the striatum has attracted attention related to DA, but the insula may be important as well. The insula has gained attention in the field of addiction because studies have shown that damage to the insula abolishes nicotine addiction (80). In an MIDT study (77), GD patients, compared with the HCs, showed decreased insular activation despite the comparable striatal activation during reward anticipation. The original RDS hypothesis supposes that addiction patients show decreased dopaminergic neuron activity, and they try to compensate for that activity with more dopamine-releasing activity (i.e., addiction-specific activity) (4). In contrast, the RDS hypothesis can be applied to patients with GD by insular activation rather than striatal dopaminergic activity. A strong DA innervation in the insula, as shown by a post-mortem study (81), anatomical connexions between the insula and striatum (82), and the lower insular activation of GD patients during reward anticipation (77), may imply a hypodopaminergic state in the insular cortex of GD patients. A resting-state fMRI study also suggested an insular role in gambling disorders. Tsurumi et al. (83) examined the resting-state functional connectivity (rsFC) between the insula and default mode network (DMN) regions (83). GD patients showed greater insular-DMN connectivity strength than the HCs (Figure 9). The insula is assumed to have a role in switching between large-scale brain networks (i.e., central executive network and DMN), leading to a shift in attention between external stimuli and internal thoughts. This switching seems to be impaired in GD patients and can result in daydreaming gambling-related thoughts even at work. Furthermore, the altered insular activation and rsFC showed correlations with the duration of illness; thus, a longer duration of illness was associated with worse functional impairment. Longitudinal studies are needed to examine the effect of the course of illness, and insular functional impairment found in GD patients (77, 83) may be an after-onset effect.

## Excessive Exercise and Supplement Use as Potential Risks for Cross-Addiction

Exercise is widely known to have health benefits for the body and the mind. However, it may potentially damage our health if has excessive intensity, frequency, and duration. Therefore, excessive exercise could be regarded as a “behavioural addiction” from a psychiatric point of view (84, 85). Excessive exercise is thought to be associated with psychological states such as anxiety about self-appearance, body weight, and muscle mass, as well as interest in physical and mental health. Considering the various sports disciplines, investigating the differences in the relationships between addictive tendencies for each discipline and other related psychological states including appearance anxiety (84) and self-compassion (42) among sports disciplines, may allow for the discovery of tailor-made ways to address the risk of excessive exercise for each sport discipline.

**Figure 10:**
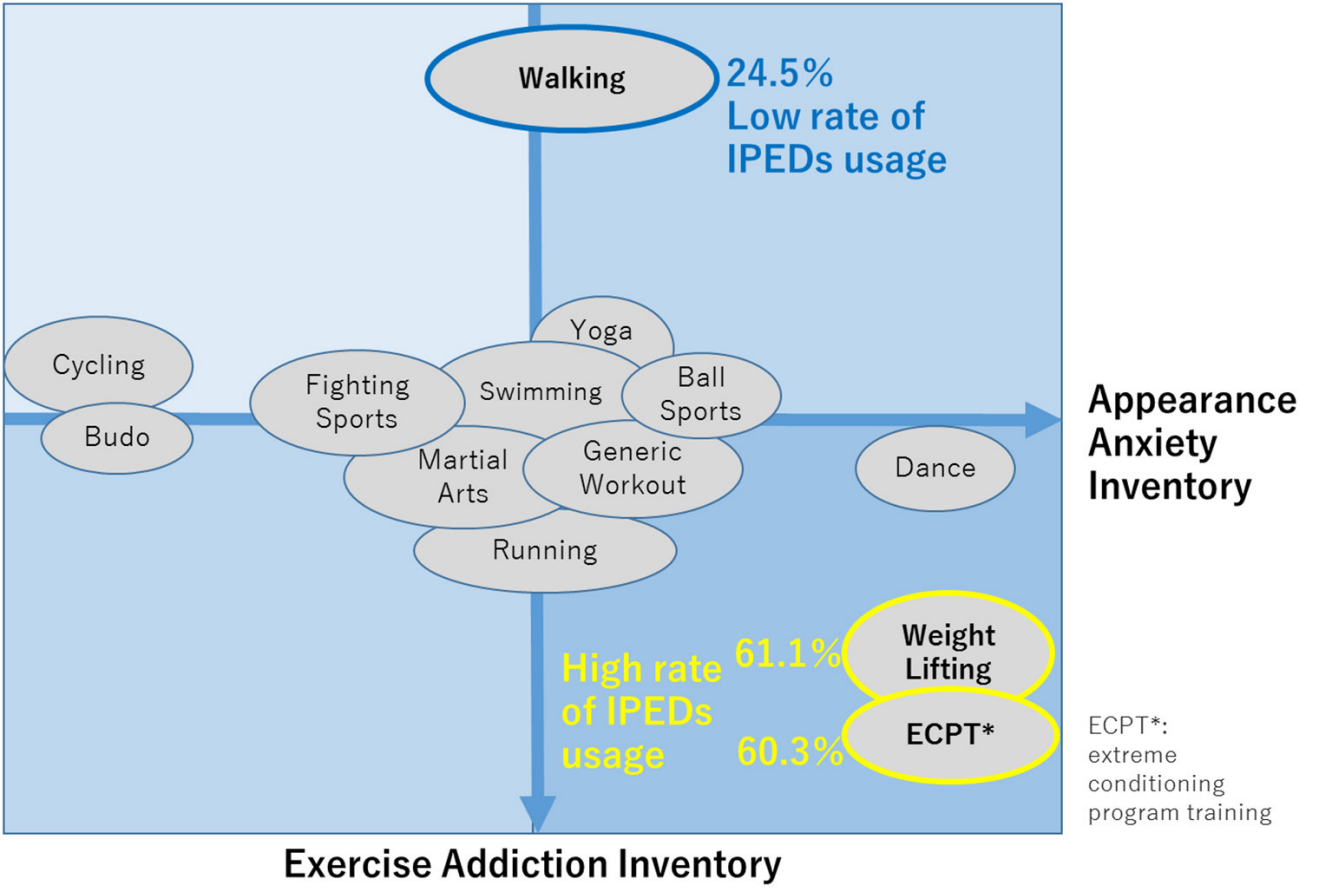
Characteristics of each disciplines according to EAI and AAI scores. EAI, exercise addiction inventory; AAI, appearance anxiety inventory [Revised from Shibata et al. (86)].

Shibata et al. (86) investigated the relationship between excessive physical exercise and abuse of image and performance-enhancing drugs (IPEDs), which are commonly used by athletes and potentially result in a form of over-enhancement, such as doping, among those who habitually engage in sports, considering the concept of “cross-addiction”, the state in which each comorbid perpetuates each of the addictions (86). Starting in 2020, our lifestyle, including exercise habits, has changed dramatically due to the coronavirus disease 2019 (COVID-19) pandemic. In this study, the differences in sports disciplines related to the potential risk of excessive engagements and their association with IPED use during COVID-19 pandemic. Regarding the risk of excessive physical exercise associated with each sport discipline, endurance discipline players, such as fitness centre attendees, power discipline players, and ball game players, have already shown a higher risk in the before COVID-19 pandemic situation (87). An online survey was conducted (N= 2,295), during which participants were engaged in various sports, including generic workouts, walking, weightlifting, running, yoga, fighting sports (e.g. boxing, kickboxing), swimming, dance, martial arts, cycling, ball sports, Japanese martial arts Budo, and extreme conditioning program training, hereinafter called “ECPT”. “Martial Arts” meant oriental (non-Western cultural style) fighting sports such as Judo, Taekwondo, Kendo, Aikido, Brazilian jiu-jitsu, Karate, Muay Thai, Tai Chi, Wushu, and Capoeira. “Budo” corresponded to Japanese-origin martial arts, such as Judo, Kendo, Aikido, and Karate. A summary of the results is as follows. The Exercise Addiction Inventory (EAI) (higher scores indicate greater addictiveness to exercises) score for walking was considerably lower than those for other sport disciplines, while weightlifting and ECPT had higher EAI scores than the others. The appearance anxiety inventory (AAI) scores (higher scores were associated with greater anxiety towards self-images) for Budo and cycling were lower than those for other sports disciplines, while those for weightlifting, ECPT, and dance were higher than the others (Figure 10). Regarding the difference in self-compassion scale (higher scores are associated with greater empathic concern for both self and others, which potentially leads to better stress coping) among disciplines, the score for cycling was higher than those for the others.

IPED use was significantly higher for weightlifting (61.1%) and ECPT (60.3%) but lower for walking (24.5%) than for other disciplines. EAI and AAI were positively associated with IPED use among individuals with habitual exercise. As indicated in Figure 10, the sports were generally categorised into the group with high EAI, low EAI, and others, according to the EAI scores. Weightlifting and ECPT belonged to the group with high EAI and walking belonged to the low EAI group, indicating that these results are consistent with a previous report (87) that indicated that endurance athletes, those engaged in power disciplines, fitness centre attendees were “at risk” of excessive physical exercise.

Considering the association between EAI and IPED use for these disciplines, a higher EAI was associated with greater IPED use, indicating that excessive exercise leads to the risk of “cross-addiction” with substance use, which may lead to excessive enhancement like doping. Given the increased online accessibility (12.2% of those who use IPEDs purchased them online), the risk of “triplet” cross-addiction, including excessive Internet use and excessive exercise and IPED abuse, may be considered, together with the commonality among these issues in terms of the need for regulation. In this study, professional athletes were excluded from participation to investigate the relationship between exercise habits and IPED use in non-professionals. In future studies, both professional and non-professional athletes should be investigated and compared, considering the differences in exercise levels such as intensity, frequency, and skills. In particular, the personal need of individuals for exercise in their lives (i.e., the importance and value of exercise to each individual) would vary; hence, terms such as “excessive” and “addictive” could be cautiously defined by considering the entire body of evidence non-professional and professional athletes. Future studies should consider adequate DA neurotransmission (regulation of the reward system as a consequence) for establishing optimised strategies for dealing with these habits in future studies. Interestingly, the self-compassion scale score was significantly higher, and the EAI and AAI scores were relatively lower for cycling than for other disciplines. In this context, high self-compassion may have contributed to the lower EAI and AAI scores for cycling. These health benefits may not be directly interpreted as cycling-specific, and “mind-body” integrated training programs for different types of sports, in general (which potentially foster self-compassion), may regulate DA neurotransmission, leading to the improvement in coping skills or reducing the risk of addiction as a consequence.

## Discussion

The possibility of “optimal, hyper-normal” level behaviours and future directions are discussed below: The previous reports introduced in this current review are from cross-sectional comparisons, and several interpretations of the causality of habitual behaviours and mental health would be possible. (1) Considering the low to intermediate media multitasking and IU as a part of the background of the multitasking, these may be potential mental/cognitive health benefits, as long as the level is not excessive. (2) A homeostatic attention-related network connectivity is assumed to be a possible interpretation of fewer changes in network FCs in individuals with higher resilience and low to intermediate levels of media multitasking. (3) Habitual Budo training, which closely overlaps with Zen meditation, can help foster the motivation that can partially influence its potential benefits related to the cultivation of self-compassion as “Zen in action”. (4) Habitual physical exercise has health benefits; however, the risk of related addictive problems should be considered in the same light as other habitual behaviours, and its potential to lead to cross-addiction with substance misuse should not be overlooked.

Regardless of differences in interpretation, there are several limitations to each study cited in this article. First, the studies in which the positive benefits of each behaviour were discussed (i.e., IU, multitasking, and Kendo as an exercise) were conducted using a cross-sectional design. Therefore, caution should be exercised when discussing the causality between life habits (behaviours) and the changes in cognition, including those related to the reward system. Future studies with a longitudinal design would be needed to address this point. Second, the sample sizes were relatively small, particularly in the study of the benefits of Budo on motivation (54). Similarly, in the study of exercise addiction tendency and IPED use (86), the number of participants in the subgroups (e.g., those with exercise addiction levels exceeding the cut-off) for each sports discipline was small, despite the total number of the participants being acceptable. The same investigation should be conducted using a larger sample size. Finally, in the papers regarding IU (29), Budo (54), multitasking (39), and resilience (60), the level of cognitive loads/demands of the paradigm (oddball task) is very low for attention processing. Therefore, incorporating paradigms with different degrees of difficulty into future studies would lead to a better understanding of the essential meanings of these themes.

**Figure 11:**
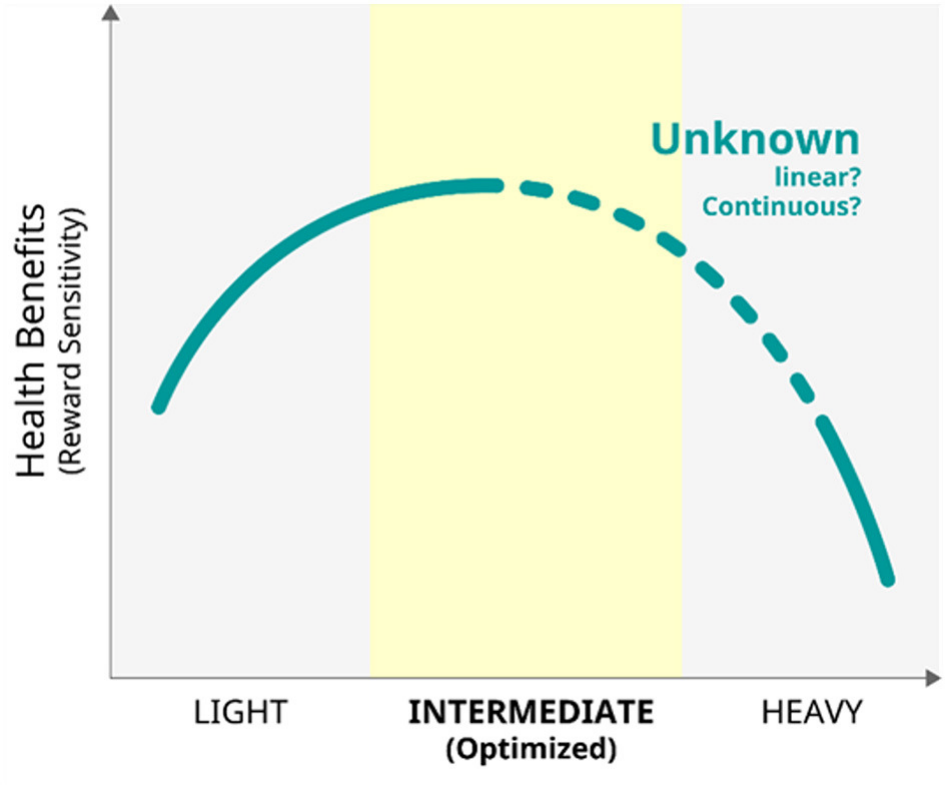
The model of the relationship between habitual behaviours and health benefits of the reward system.

Habitual behaviours introduced in the current review cannot be avoided in our social lives in this era. Gambling is not always necessary in daily life; however, it is still a form of entertainment for many people when engagement is not excessive (for example, as found in the advertisements of casinos). It is important to monitor and estimate the level of each behaviour and other related psychological backgrounds to better understand the optimal frequency, intensity, and duration of engagements for each behaviour. Direct comparisons of various levels of multitasking revealed that intermediate multitasking requires the most attention processing, suggesting an inverted U-shape association between multitasking tendency and attention function (88). In this context, the inverted U-curve model may be generalised to other habitual behaviours (Figure 11). For other aspects, it is important to recognise that each individual generally engages in different daily habits, that is, just like multitasking itself. For example, PUI includes several activities, such as net surfing, online shopping, online pornography, social media-related activities, and online gaming. Therefore, each activity should be investigated separately; participants with various levels of engagement, including light, moderate, and heavy levels, should be recruited, and common problems should be extracted from, those issues. In this sense, the indices of the reward system, which is modulated by DA neurotransmission, may be used as biomarkers to clarify the best strategies in dealing with each habitual behaviour and imaging the best way of life.

## Author Contributions

KT, MS, KK, TMi, NO, TMu, TU, and HF wrote, edited, and supervised the manuscript. All authors contributed to the article and approved the submitted version.

## Funding

This study was supported by Grant-in-Aid for Scientific Research (B) (Japan Society for The Promotion of Science, 21H02849), Grant-in-Aid for Transformative Research Areas (A) (Japan Society for The Promotion of Science, JP21H05173), Grant-in-Aid by the Smoking Research Foundation, Grant-in-Aid for Scientific Research (A) (Japan Society for The Promotion of Science, 19H00518), The Strategic International Brain Science Research Promotion Program (Brain/ MINDS Beyond) (21dm0307102h0003) from Japan Agency for Medical Research and Development (AMED).

## Conflict of Interest

The authors declare that the research was conducted in the absence of any commercial or financial relationships that could be construed as a potential conflict of interest.

## Publisher's Note

All claims expressed in this article are solely those of the authors and do not necessarily represent those of their affiliated organizations, or those of the publisher, the editors and the reviewers. Any product that may be evaluated in this article, or claim that may be made by its manufacturer, is not guaranteed or endorsed by the publisher.
